# Development and Psychometric Properties of the Pressure Injury Prevention Knowledge Questionnaire in Spanish Nurses

**DOI:** 10.3390/ijerph17093063

**Published:** 2020-04-28

**Authors:** María Dolores López-Franco, Laura Parra-Anguita, Inés María Comino-Sanz, Pedro L. Pancorbo-Hidalgo

**Affiliations:** Department of Nursing, Faculty of Health Sciences, University of Jaén, 23071 Jaén, Spain; lparra@ujaen.es (L.P.-A.); icomino@ujaen.es (I.M.C.-S.); pancorbo@ujaen.es (P.L.P.-H.)

**Keywords:** knowledge, prevention, surveys and questionnaires, nursing staff, validation studies, pressure ulcer

## Abstract

Pressure injuries (PIs) are a major health problem with severe implications for patients. Professionals who care for people at risk should have high knowledge about PIs prevention. The actual knowledge can be measured using different tools, but we have found no questionnaire to measure the knowledge on PIs prevention developed and validated for Spanish-speaking countries. The aim of this study was to develop a questionnaire in Spanish to measure the knowledge about PIs prevention based on current international guidelines. Content validity was evaluated by 12 experts in wound care. A convenience sample of 438 nursing professionals from Spain participated to evaluate the questionnaire using item analysis, Rasch model, and known-groups validity. The PI Prevention Knowledge (PIPK) questionnaire shows good discrimination and difficulty indices. The 31-item PIPK shows good fit and reliability of 0.98 for items and 0.72 for people; also, it has enough evidence for construct validity. Because the questionnaire has been developed based on the recommendations from international guidelines, the English version of this questionnaire could be used in further studies to test its psychometric properties.

## 1. Introduction

Pressure injuries (PIs) are a major health problem with severe implications for patients [1], for institutions because of the high costs for treatment [2,3], and also for health professionals because they are considered healthcare-related adverse events [4]. In many countries, there are also legal issues because PIs are considered as caused by inadequate care and compensation for patients who developed PIs at hospitals has been as high as $312 million [5].

The prevalence of PIs has a lot of variation across different countries and settings around the world. For European countries, some recent epidemiological studies reported a PIs prevalence of 11.7% in Germany (in both hospitals and nursing homes) [6], 24.2% in Sweden [7], 14.9% in hospitals in Norway [8], 7% in hospitals in Spain [9], 22.7% in acute hospitals in Italy [10], and 27% in long-term units in Italy [11]. If intensive care units (ICU) were included, then the prevalence could be as high as 54% [12]. For patients at hospitals, most of the PIs were acquired during the stay; it has been reported that up to 72.2% of these injuries are hospital-acquired [9].

In Australia, the reported prevalence ranged from 3% in inpatient wards to 11.5% in ICUs [13]. In Brazil, the prevalence of PIs was 40% in an emergency hospital unit [14] and 18.8% in oncologic patients receiving home care [15]. For long-term care facilities, a recently conducted review reported prevalence rates ranging from 3.4% to 32.4% [16].

There are several factors involved in the development of PIs in at-risk patients, usually grouped into intrinsic factors (e.g., nutrition, age, immobility, and skin ischemia) and extrinsic factors (e.g., pressure, shear, and moisture) [17]. Prevention is the best approach for PIs, and a set of recommendations have been established in guidelines; briefly, risk assessment, skin assessment, preventive skin care, nutrition treatment, repositioning and early mobilization, and use of support surfaces to relieve the pressure [18]. For an adequate implementation of theses preventive measures, it is of paramount importance that health providers, especially nurses, have a positive attitude and high knowledge about the assessment and management of PI [19].

Some authors have pointed out that poor knowledge about PIs prevention in health professionals can lead to misconceptions and bad patient outcomes [20]. The actual knowledge can be measured using different tools; a recent review found up to seven questionnaires and scales for this that have some extent of validation [21]. From them, there are two questionnaires that have been used and tested in several studies: the Pieper Pressure Ulcer Knowledge (PPKUT) questionnaire [22] and the Pressure Ulcer Knowledge Assessment Tool (PUKAT) questionnaire [20]. Recently, updated versions of these questionnaires have been published, the Pieper-Zulkowski Pressure Ulcer Knowledge test (PZ-PKUT) [23] and the PUKAT 2.0 [24]. 

However, in the literature review, we found no questionnaire to measure the knowledge on PIs prevention developed and validated for Spanish-speaking countries, which was updated according to the current recommendations of guidelines. A research project conducted by our team, named “Pressure injuries as adverse events: patient safety issues, knowledge, attitudes and perceived barriers by nurses in Spain” (SECOACBA project) addressed this gap. The main goals of this project were: (a) To determine the degree of knowledge, the perception of patient safety, the attitudes, and the perceived barriers that nurses have to PIs prevention in hospitals in Spain; (b) To explore whether there is any association between knowledge, perception of patient safety, attitudes, and perceived barriers to the prevention of PIs. The first step was to develop the measuring tools, so one of the specific aims of this project was to develop an updated questionnaire to measure the knowledge about PIs prevention based on current international guidelines and to test its psychometric properties. This article reports on this aim.

## 2. Materials and Methods

### 2.1. Design

The new questionnaire was developed through three stages of research:

- Development of the questionnaire and item wording.
- Content validation by an expert panel.
- Evaluation of the psychometric properties through an observational study in four hospitals. The period of data collection was from March to April 2017.

### 2.2. Questionnaire Development

Starting with 7 guidelines for PIs prevention published since 2009 [25,26,27,28,29,30,31], a total of 414 recommendations were extracted. After grouping and deleting duplicates, 84 recommendations on PIs prevention were obtained. From these recommendations, we wrote our first version of the knowledge questionnaire with 52 items. 

### 2.3. Content Validation

Content validation of items was carried out by a panel of 12 experts in wound care, through three consecutive rounds. The experts were members of the Spanish National Advisory Group on Pressure Ulcers and Chronic Wounds; all of them were nurses with more than 10 years of experience working in hospitals (7), primary care (3), and universities (2).

Each expert was asked to rate the relevance and clarity of each item, using a 5-point scale from 1 (not relevant-not clear) to 5 (very relevant-very clear). We used the V-Aiken index and 95% confidence interval [32] to establish consensus among experts using the value of 0.80 as the threshold for retaining the items.

From the first version of the questionnaire with 52 items, after the first round with an expert panel, 33 item were accepted, 10 items removed, and 9 items modified and moved on to the second round.

In the second round, 4 out of the 9 items were retained, obtaining a version of the questionnaire composed of 37 items (version 2).
In 2017, some items were revised and reworded, so the questionnaire was submitted to a third round with the expert panel; 2 items were removed, yielding a 35-item version of the PIPK questionnaire (version 3) that was used for testing the psychometric properties.

### 2.4. Psychometric Testing: Population

The study was carried out in 4 public hospitals (2 acute, 1 mother and child, and 1 long-term care) from the University Hospital of Jaén (Jaén, Spain). These hospitals belong to the Andalusian Health Service and were public funded. 

The population surveyed were Registered Nurses (RNs) and Assistant Nurses (ANs) with more than 6 months of clinical experience, working in one of these hospitals. An “a priori” minimum sample size of 175 people was estimated as necessary, according to the methodological recommendations for the validation of questionnaires (5 people per item) [33]. However, to maximize the sample size, all the RNs and ANs working in 29 units of the hospitals were invited to participate and complete the questionnaire.

### 2.5. Data Collection

Demographic and educational characteristics of the participants were collected by a specific form. The 35-item version of the PIPK questionnaire was self-administered (paper version). The questionnaire had 35 items with statements about prevention and three answer options: “True”, “False”, and “I don’t know”. The “I don’t know” option was included to allow respondents to indicate their ignorance. For 21 items, the correct answer was “True”, and for 14 items, the correct answer was “False”.

The Nursing Director of the University Hospital authorized the study, and all the unit managers were informed and asked to collaborate. 

The self-administered questionnaire was designed by a person with experience in carrying out surveys with a specific format to be read by an optical reader. In this way, the questionnaires were read, validated, and evaluated without human intervention. The questionnaires were provided with an envelope and an explanatory document informing about the purpose of the study. Consent to participate was given if nurses handed in the questionnaire in the sealed envelope. For the collection of the questionnaires and to guarantee anonymity and confidentiality, each unit was provided with a box to collect the sealed envelopes. 

### 2.6. Data Analysis

The data obtained were tabulated, coded, and cleaned in a spreadsheet before the analysis. The methods used for the analysis were: item analysis, Rasch model, and known-groups validity.

#### 2.6.1. Item Analysis

For each item of the questionnaire, three indices were calculated: discrimination, ignorance, and correctness. The discrimination index was the difference between the percentage of correct answers for the 27% of questionnaires with the highest score minus the 27% of questionnaires with the lowest score [34]. Items were classified into 4 categories based on this index: good (>0.30), moderate (0.20–0.29), low (<0.20), and bad (negative values). The ignorance index was defined as the percentage of “I don’t know” answers. The correctness index was defined as the percentage of correct answers. Items were classified into five categories: very easy (≥90%), easy (75%–89%), medium (50%–74%), difficult (25%–49%), and very difficult (<25%).

#### 2.6.2. Rasch Model

The mathematical model proposed by George Rasch uses a probabilistic model to measure and calculate latent and unobservable traits. This model can measure a person’s ability according to the probabilities of obtaining a correct answer to a particular item in a given test or questionnaire. The Rasch model has the advantage of measuring both people and items in a single dimension and estimating the relationship between the ability of respondents and the difficulty of the items [35,36,37]. The Rasch analysis was conducted in Jmetrik software [38]. For parameters estimation, we used the joint maximum likelihood estimation method [38]. The fit of the model was estimated by the unweighted mean square of standardized residuals (UMS) and the weighted mean square of standardized residuals (WMS). Values of fit indices between 0.8 and 1.2 mean good fit, and values between 0.5 and 1.5 mean acceptable fit. The assumption of local independence between items was tested among the items using Yen’s Q3 statistic [39]. 

Differential item functioning analysis (DIF) allows to identify the items that have different responses in different groups. This is a statistical characteristic of an item that shows the extent to which the item might be measuring different abilities for members of separate subgroups. For this technique, two groups have to be compared (denominated focal and reference group) without any special criterion, the only important thing is to clearly identify which is each of these groups [40]. DIF analysis was done by comparing the RNs group (focal group) with the ANs group (reference group). The effect size (common OR) and 95% confidence interval were calculated. An item was considered to have no differential functioning when the common OR had a value between 0.65 and 1.53. Items with an effect size <0.53 or >1.89 were considered to have a large DIF, and those between 0.53 and 0.65 or 1.53 and 1.89 were considered to have a low DIF [40].

#### 2.6.3. Validity

Criterion validity was not tested, because there is no gold standard (a tool for measurement of PIs prevention knowledge validated in Spanish). Therefore, we used the known-groups test as an alternative, which is based in the comparison of one group with expected high knowledge versus one group with expected low knowledge [41]. Criteria to build these groups were professional category and specific training on PIs prevention.

The hypotheses tested were:

- Higher knowledge score in the RNs group than in the ANs group.
- Higher score in professionals who have received specific training on PIs prevention compared to those who have not received specific training.

### 2.7. Ethics

The research project was approved by the Committee of Research Ethics of Jaén. The data obtained were anonymous according to the Spanish Law of Personal Data Protection. On the first page of the form, participants were fully informed about the purpose and procedures of the study, and it was stated that the completion and submission of the questionnaires implies an agreement to participate.

## 3. Results

### 3.1. Content Validation

After the first round with the panel of experts, 33 items were accepted, 10 items removed, and 9 items modified and moved on to the second round. In the second round, 4 out of the 9 items were retained, obtaining a version of the questionnaire composed of 37 items (version 2). Some items were revised and reworded, so the questionnaire was submitted to a third round with the expert panel; 2 items were removed, yielding a 35-item version of the questionnaire (version 3) that was used for testing the psychometric properties. This new developed questionnaire was named the Pressure Injury Prevention Knowledge (PIPK) questionnaire.

### 3.2. Sample Characteristics

A total of 438 nursing professionals (RNs and ANs) from 29 units (inpatient wards and ICUs) participated in the study, with a response rate of 50.8%. Table 1 shows the main characteristics of this sample.

### 3.3. Item Analysis

Initially, the indices of discrimination, correctness, and ignorance were calculated from the 35-item version. For the correctness index (percentage of correct answers), there were 17 items very easy, 8 easy, 4 medium, 4 difficult, and 2 very difficult.

The discrimination index indicated that 19 of the items had a good discrimination, with 10 moderate, 5 low, and 1 bad. Items with low and bad discrimination indices were marked as possible candidates for elimination after the Rasch analysis. 

The item “In dark-skinned patients, skin assessment should prioritize skin temperature, presence of oedema and change in tissue consistency instead of the appearance of erythema” obtained a higher percentage of “I don’t know” answers (13%).

### 3.4. Rasch Model

Local independence of the items was confirmed with Yen’s Q3 statistic, obtaining a residual correlation >0.20 in only 5 cases of the 961 correlations of the model. Rasch analysis of the 35-item version indicated that 4 items (two of them previously identified with the discrimination index) had a poor fit to the model (values >1.5). These four items were: “Patients with reduced mobility can be seated over a standard cushion for comfort” (false); “Visual examination of the skin is sufficient to identify category I pressure ulcers” (false); “The information given to patients and caregivers about pressure ulcers must include: causes and early symptoms, ways to prevent, consequences of having an ulcer and a demonstration of techniques and equipment for prevention” (true); and “Clean the skin as soon as possible after each episode of urinary or faecal incontinence” (true). 

These items were removed and a new Rasch analysis was done for the 31-item version. Table 2 shows the fit indices and difficulty score of each item. All the items fit well in the WMS (only item 9 slightly exceeds 1.5, but fits well with the UMS).

**Figure 1:**
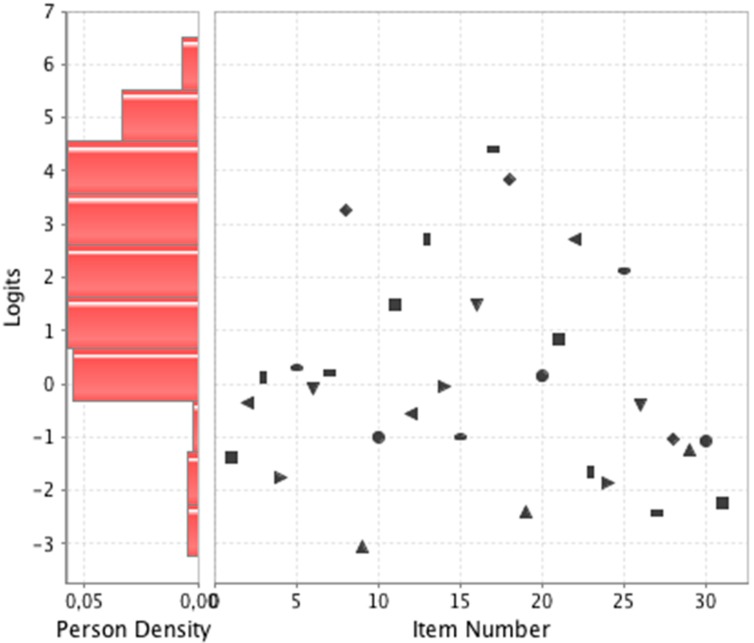
The items map of the PIPK questionnaire. Person density indicates the distribution of scores obtained by people (expressed in logit units). Values higher than 0 indicate high knowledge, and values lower than 0 indicate low knowledge. The points on the graph indicate the distribution of the items according to their difficulty. The horizontal axis shows item number (ordered from 1 to 31), and the vertical axis shows the difficulty index of the items, where higher values indicate more difficult items.

Additionally, the items of the PIPK questionnaire had a wide range of difficulty, from the easiest (item 9 “In bedridden patients at risk of pressure injuries, a mattress with pressure-relieving properties should be used instead of a standard mattress” (true), difficulty: –3.07) to the most difficult (Item 17 “Protect the skin from moisture by applying hyper-oxygenated fatty acids” (false), difficulty: 4.40). The items map (Figure 1) showed that the distribution of the scores obtained by people and the difficulty index of items matches, meaning that the questionnaire performs well to measure the knowledge. Overall, the PIPK questionnaire has good quality indices and high reliability (Table 3). 

### 3.5. Differential Item Functioning

The analysis revealed that no item had a large DIF, and only four items had a small DIF, performing differently in RNs and ANs. Two of the items (2 and 8) performed better in the RNs compared to the ANs group, and 2 of the items (9 and 13) performed better in the ANs group. Overall, DIF analysis showed that the questionnaire was suitable for use in both groups (Table 4). 

**Figure 2:**
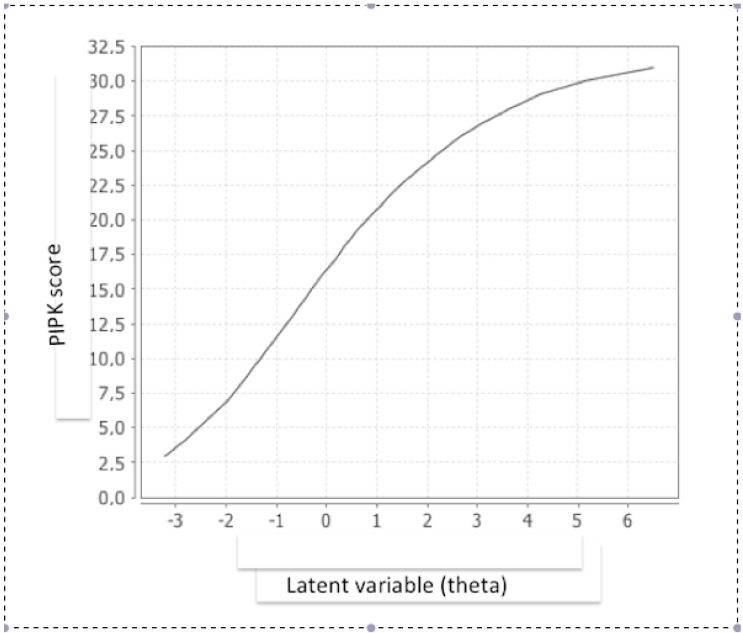
Graphic of the direct score of the PIPK questionnaire (Y axis) versus the true score of the latent variable measured (theta) (X axis).

Figure 2 shows the curve of the scores obtained in the questionnaire against the values of the latent variable (theta score) estimated by the model. The S shape of the upper part of the curve means that small increases in score suppose larger increases in the latent variable measured (actual knowledge of PI prevention).

### 3.6. Construct Validity

Construct validity was confirmed by testing known-groups hypotheses. Those groups with “a priori” greater knowledge of PIs prevention obtained higher scores with the PIPK questionnaire. RNs scored higher than ANs, and nurses that had received specific training on PIs prevention also scored higher than those without this training (Table 5).

### 3.7. Pressure Injuries Prevention Knowledge (PIPK) Questionnaire

The final version of the new developed tool, the PIPK questionnaire, after the process of content validation and testing the reliability and validity is displayed in full in Table 6. The text of each item is shown in both English and Spanish; thus it may be used for international researchers. For each item, the key for the correct answer is indicated between brackets (True/False).

## 4. Discussion

The purpose of this study was to develop a questionnaire to measure the knowledge on PIs prevention with good psychometric properties and useful in the Spanish-speaking context, but also in the international context. The PIPK questionnaire has 31 items that explore the knowledge about the recommendations extracted from current PIs prevention guidelines for RNs and ANs. For this study, we defined “knowledge on PIs prevention” as the amount of knowledge that nurses and other healthcare providers have on the correct recommendations to avoid the appearance of PIs. 

The content validity of the PIPK questionnaire was evidenced by the use of robust methods: a large enough number of experts in the panel (12) and 3 successive rounds. This was recommended in order to achieve a solid consensus [42] and was the method used to develop other questionnaires [24,43]. By contrast, some published questionnaires had a small number of experts in the validation panel [22,44], which reduces the robustness.

To our knowledge, this is the first time that Item Response Theory and Rasch analysis have been used to develop a PIs questionnaire. The evaluation of the psychometric properties of PIs knowledge questionnaires published was done by methods based on the classical test theory, such as PZ-PKUT [23] and PUKAT 2.0 [24]. The main advantage of the Rasch analysis is that this method allows to estimate the error of measurement for each item, to estimate a score for the latent variable, and to measure reliability of both the items and persons. Also, it is possible to measure the difficulty of each item and match it with the person’s ability to answer the questionnaire through an items map. For the PIPK, our data show high reliability for items and persons, without the significant variability reported in other studies [45,46]. Furthermore, the range of difficulty of the items in this questionnaire seems to be quite adequate to distinguish between people with low or high knowledge [47].

This questionnaire works equally well to test the knowledge about PIs prevention of nurses from different services and with different education levels, from ANs to RNs. This is an important feature of the PIPK questionnaire, which we want to highlight because all nursing staff must be involved in PIs prevention. However, the questionnaire has not yet been tested on other healthcare provider groups, such as doctors, paramedics, informal caregivers, or nursing students. This opens up some opportunities for future research to test whether the questionnaire could be used in all these groups.

There is some controversy about adding the answer option “I don’t know” in knowledge tests, besides the options “True/False” or “Yes/No”. Some authors advise including this option to avoid random correct responses and to detect lack of knowledge on the topic being measured [48,49]. Following these recommendations, the PIPK questionnaire includes an “I don’t know” option for each item that allows for the identification of specific topics about which nurses have poor knowledge. There are some questionnaires that present this option [22,23,24,50], but most of the published PIs knowledge questionnaires do not have it.

The items about non-recommended measures, whose correct answer is “False”, are among the least known. Our analysis shows that the five items in the PIPK questionnaire with a high difficulty index and a high percentage of errors and “I don’t know” answers, refer to non-recommended measures. These were items 8 (cotton bandage for protecting heels), 13 (delaying the first risk assessment by more than 24 h), 17 (using hyper-oxygenated fatty acid to protect from moisture), 18 (keeping the head elevated more than 30º in bed), and 22 (using a doughnut-shaped device on the coccyx). This fact highlights the importance of including some statements about non-recommended interventions among the items in the questionnaire in order to truly evaluate the knowledge of nurses, because some of these incorrect measures have been applied in practice by tradition, despite being outdated.

The construct validity of the PIPK questionnaire was analyzed by the known-groups method. Overall, the questionnaire worked as expected, as nurses identified as experts in PIs prevention scored higher on the questionnaire. This is evidence of validity for the PIPK questionnaire that is consistent with findings reported in other studies [20,22,24,51]. However, convergent validity with another well validated questionnaire, as a gold standard, was not analyzed in our research. The main reason was the lack of a questionnaire that could be considered as a gold standard in the Spanish context. As described in the introduction, there are several questionnaires aimed at measuring PIs prevention knowledge, but all of them were developed and tested in English contexts. As a result, they are not directly usable in our context. Further research to test the validity of this questionnaire is warranted, not only at hospitals but also in nursing homes and in community care.

This study has several limitations that need to be recognized. During the validation of the questionnaire, the temporal stability of the questionnaire was not tested by the test-retest method, nor the convergent validity with a gold standard tool, as stated above. Although we think that the validation procedure was sound enough, these aspects of reliability and validity need to be tested in further studies. The sample of nurses used in this research was not random but composed of those individuals who accepted to participate in the survey, so it is possible that they were nurses more motivated or interested in PIs prevention, which could have resulted in a bias in the score of knowledge obtained. Finally, the study was conducted in hospitals, so the evidence about the psychometric properties of the questionnaire can be applied for hospital nurses but not for other settings, such as nursing homes or community care. Studies are needed to test the performance of the PIPK questionnaire in different clinical settings and populations to broaden its applicability.

## 5. Conclusions

The PIPK questionnaire is a useful tool to measure the knowledge about PIs prevention of nurses. The Spanish version offers evidence of reliability and validity in the hospital setting. Because the questionnaire has been developed based on the recommendations of international guidelines, the English version of this questionnaire could be used in further studies to test its psychometric properties.

By using this questionnaire, a global score of knowledge on PIs prevention can be obtained, but it is also possible to identify specific points of prevention that the professionals ignore or have misunderstandings about.

This questionnaire is a versatile tool that can be used both in clinical practice and by managers to assess gaps in knowledge about prevention among professionals working with people at risk of developing PIs, which is considered to be an adverse event related to patient safety.

## Figures and Tables

**Table 1:** Socio-demographic characteristics of the Registered Nurses and Assistant Nurses.

Variable	Frequency ^1^ (%)
Gender	
Female	354 (80.8)
Male	52 (11.9)
Age (years)	
20–30	7 (1.6)
31–40	57 (13.0)
41–50	162 (37.0)
51–60	192 (43.8)
61–69	16 (3.7)
Professional category	
Registered nurse	266 (60.7)
Assistant nurse	161 (36.8)
Academic degree	
Technical training (2 years)	150 (34.2)
Nursing diploma (3 years)	228 (52.1)
Nursing degree (4 years)	27 (6.2)
Bachelor (4 years)	7 (1.6)
Postgraduate: Master	15 (3.4)
Doctorate	2 (0.5)
Work experience (years)	
<10	30 (6.8)
11–20	124 (28.3)
21–30	176 (40.2)
>31	104 (23.7)
Specific training in prevention of PIs	
None	67 (15.3)
Basic ^2^	93 (21.2)
Multiple ^3^	278 (63.5)
Uses protocol	
Yes	253 (57.3)
No	174 (39.7)
Engagement in research on PIs	
Yes	59 (11.9)
No	378 (86.3)

^1^ The percentage missing from the variables up to 100% corresponds to the “No” answers. ^2^ Include only basic or undergraduate. ^3^ Multiple training: basic plus conference attendance and/or continuous training.

**Table 2:** Rasch model for the Pressure Injury Prevention Knowledge (PIPK) questionnaire with 31 items.

Item	Difficulty (Standard Error)	WMS (Infit)	UMS (Outfit)
1	−1.40 (0.26)	1.04	1.31
2	−0.36 (0.18)	1.05	1.57
3	0.11 (0.16)	1.15	1.38
4	−1.77 (0.31)	1.18	1.53
5	0.31 (0.15)	1.00	0.89
6	−0.09 (0.17)	0.91	0.78
7	0.22 (0.15)	0.97	1.00
8	3.25 (0.11)	1.05	1.13
9	−3.07 (0.51)	1.56	1.15
10	−1.00 (0.23)	1.12	1.15
11	1.48 (0.12)	0.90	0.85
12	−0.56 (0.19)	0.86	0.72
13	2.72 (0.11)	1.03	1.32
14	−0.04 (0.16)	0.99	0.91
15	−1.01 (0.23)	0.86	1.25
16	1.50 (0.12)	1.14	1.32
17	4.40 (0.14)	1.05	3.68
18	3.83 (0.12)	1.05	4.06
19	−2.40 (0.39)	1.12	0.92
20	0.14 (0.15)	1.03	0.97
21	0.82 (0.13)	0.90	0.80
22	2.71 (0.11)	1.01	2.23
23	−1.65 (0.29)	0.94	0.87
24	−1.86 (0.32)	0.80	0.36
25	2.12 (0.11)	1.12	1.15
26	−0.40 (0.18)	1.20	2.08
27	−2.41 (0.39)	1.02	1.67
28	−1.03 (0.23)	1.13	0.84
29	−0.40 (0.18)	1.16	1.55
30	−2.41 (0.39)	1.08	0.57
31	−1.03 (0.23)	0.76	0.27

* WMS: Weighted mean square of standardized residuals; UMS: Unweighted mean square of standardized residuals.

**Table 3:** Statistics for the PIPK questionnaire with 31 items.

Statistic	Items	Persons
Observed variance	3.64	1.55
Adjusted variance	3.58	1.12
Separation index	8.08	1.61
Number of strata	11.11	2.48
Reliability	0.98	0.72

**Table 4:** Analysis of differential item functioning for the PIPK questionnaire comparing RNs versus ANs.

Item	OR (IC 95%)	*p* Value	DIF
1	1.06 (0.36–2.98)		
2	0.35 (0.16–0.74)	0.01	+ (favors RNs)
3	1.46 (0.77–2.79)		
4	1.03 (0.35–3.03)		
5	0.97 (0.53–1.75)		
6	1.01 (0.50–2.02)		
7	0.62 (0.34–1.14)		
8	0.52 (0.31–0.86)	0.01	+ (favors RNs)
9	>10 (>10)	0.01	- (favors ANs)
10	2.10 (0.81–5.48)		
11	1.05 (0.63–1.74)		
12	0.95 (0.42–2.16)		
13	2.42 (1.46–4.01)	<0.001	- (favors ANs)
14	1.15 (0.59–2.25)		
15	2.75 (0.9–7.87)		
16	0.89 (0.56–1.41)		
17	0.70 (0.38–1.29)		
18	1.54 (0.90–2.64)		
19	0.49 (0.11–2.26)		
20	0.56 (0.30–1.02)		
21	1.12 (0.64–1.95)		
22	0.76 (0.47–1.23)		
23	1.34 (0.44–4.06)		
24	1.56 (0.45–5.47)		
25	0.96 (0.62–1.49)		
26	1.44 (0.71–2.92)		
27	1.37 (0.36–5.19)		
28	1.24 (0.53–2.89)		
29	1.30 (0.53–3.17)		
30	0.49 (0.19–1.29)		
31	3.19 (0.84–18.58)		

**Table 5:** Construct validity in known groups.

Variable	Mean (SD)	*p* Value
Professional category		
Registered nurses	18.01 (2.44)	< 0.0001
Assistant nurses	17.04 (2.64)	
Specific training on PIs prevention (overall score)		
None (N = 66)	17.15 (2.02)	0.001
Multiple * (N = 273)	18.01 (2.40)	
Specific training on PIs prevention (split by professional category)		
Registered nurses		
None (N = 46)	17.22 (2.01)	< 0.0001
Multiple (N = 174)	18.41 (2.18)	
Assistant nurses		
None (N = 16)	16.94 (2.20)	0.572
Multiple (N = 95)	17.27 (2.8)	

SD: Standard deviation. *p* value for groups difference with the Mann–Whitney test. * Multiple includes basic training, courses, conferences, and continuing education.

**Table 6:** The final version of Pressure Injury Prevention Knowledge (PIPK) questionnaire.

English Version	Spanish VersionCuestionario de Conocimientos Sobre Prevención de Lesiones por Presión
1. When repositioning the individual in bed, use some device or fabric to reduce friction and shear forces and avoid dragging on the bed surface. (T)	1. Al cambiar de posición al individuo, reduzca la fricción y cizalla utilizando aparatos y dispositivos auxiliares (del tipo entremetida) que impiden el arrastre sobre la superficie (V)
2. Offer high-protein, high-calorie nutritional supplements to adults at risk for pressure injuries if dietary intake does not meet nutritional requirements. (T)	2. Ofrecer suplementos nutricionales con alto contenido en proteínas y calorías en adultos con riesgo de lesiones por presión si la ingesta dietética es insuficiente. (V)
3. When repositioning in bed, patients can be placed over reddened skin areas. (F)	3. Al hacer cambios posturales, el paciente puede apoyarse sobre zonas corporales enrojecidas. (F)
4. Reassess the risk of pressure injuries when a significant change in patient health status, or clinical situation happens. (T)	4. Reevaluar el riesgo de lesiones por presión si cambia la situación clínica o de cuidados del paciente. (V)
5. Assess and monitor nutrition using some validated assessment tools, in a way appropriate to the population and clinical context. (T)	5. Realizar la monitorización y evaluación nutricional utilizando herramientas validadas, de forma adecuada a la población y entorno clínico. (V)
6. Skin areas in contact with medical devices (such as masks or tubes) do not have a higher risk for developing pressure injuries. (F)	6. Las áreas de la piel en contacto con dispositivos clínicos (sondas, mascarillas, etc) no presentan mayor riesgo de desarrollo de lesiones por presión. (F)
7. Describe all pressure injuries using a standardized classification system. (T)	7. Describir todas las lesiones por presión siguiendo un sistema de identificación estandarizado. (V)
8. A cotton and elastic bandage on the heels allows to redistribute the pressure and prevent pressure injuries. (F)	8. Utilizar algodón y venda ajustable permite redistribuir la presión sobre talones y prevenir las lesiones por presión.(F)
9. In bedridden patients at risk of pressure injuries, a mattress with pressure-relieving properties should be used instead of a standard mattress. (T)	9.En pacientes encamados con riesgo de lesiones por presión, usar un colchón con propiedades de alivio de la presión, en vez de un colchón estándar. (V)
10. The skin in contact with medical devices (such as drains or tubes) should be protected by using hyper-oxygenated fatty acids and/or foam dressings. (T)	10. Proteja la piel en contacto con los dispositivos clínicos (sondas, drenajes, etc) utilizando ácidos grasos hiperoxigenados y/o apósitos protectores con capacidad de manejo de la presión. (V)
11. Rubbing the skin with alcohol and massaging over bony prominences is useful to enhance capillary circulation. (F)	11.Masajear la piel sobre prominencias óseas o dar friegas de alcohol o colonia es eficaz para favorecer el aumento de la circulación capilar. (F)
12. It is not necessary to periodically mobilize medical devices (such as masks or tubes) to prevent pressure injuries. (F)	12. No es necesario movilizar regularmente los dispositivos clínicos (sondas, drenajes o mascarilla) para prevenir lesiones por presión. (F)
13. A comprehensive skin assessment (head to toe) of all patients admitted to a facility (hospital or nursing home) may be done within the first 48 h after admission. (F)	13. La valoración completa de la piel (de cabeza a pies) a todos los pacientes puede hacerse hasta en las primeras 48 horas tras su admisión en un centro sanitario o socio-sanitario. (F)
14. Repositioning is not necessary in bedridden patients using a pressure-relief mattress. (F)	14.En pacientes encamados que disponen de una superficie de alivio de la presión no es necesario realizar cambios posturales regulares. (F)
15. The seat tilt should be adequate to reduce pressure and shear forces on the skin in at-risk patients while sitting. (T)	15.Proporcionar una inclinación adecuada del asiento minimizando la presión y cizalla ejercida sobre la piel y tejidos blandos en aquellos pacientes que se encuentren sentados. (V)
16. In dark-skinned patients, skin assessment should prioritize skin temperature, presence of oedema, and change in tissue consistency, instead of the appearance of non-blanchable redness. (T)	16. En pacientes de piel oscura, la valoración de la piel debe priorizar la temperatura, presencia de edema y cambio de consistencia del tejido, más que enrojecimiento no blanqueable de la piel. (V)
17. Protect the skin from moisture by applying hyper-oxygenated fatty acids. (F)	17.Proteger la piel frente a la humedad mediante la aplicación de ácidos grasos hiperoxigenados. (F)
18. In at-risk bedridden patients, keep semi-incorporated with head elevated between 30º and 45°. (F)	18.En pacientes encamados, mantener semi-incorporados con cabecero de la cama elevado entre 30 y 45°. (F)
19. All risk assessments performed must be registered in the patient’s medical record. (T)	19.Documentar en la historia del paciente todas las evaluaciones de riesgo. (V)
20. Nutritional status should be assessed when the patient is admitted to a health facility or a major change in his/her health status happens. (T)	20. Evaluar el estado nutricional en caso de ingreso en un centro sanitario o un cambio significativo de las condiciones clínicas. (V)
21. Length of the surgery is not a risk factor for the development of pressure injuries. (F)	21.La duración de una intervención quirúrgica no se considera un factor de riesgo en el desarrollo de lesiones por presión. (F)
22. Use a donut-shaped device to relieve the pressure in at-risk patients with reduced mobility. (F)	22. Utilizar un dispositivo tipo “rosco” para aliviar la presión en pacientes con movilidad reducida. (F)
23. Use the most appropriate pressure relief mattress based on the patient’s characteristics, scheduling repositioning accordingly. (T)	23. Usar la superficie de alivio de la presión más adecuada en función de las características y riesgo del paciente, adaptando los cambios posturales al tipo de superficie disponible. (V)
24. In patients with incontinence, profuse sweating, wound exudation or drainage, consider the use of appropriate management devices (such as urinary catheters, diapers, or dressings). (T)	24. En caso de incontinencia, sudoración profusa, exudado de heridas o drenajes valorar la utilización de dispositivos de control adecuados (sondas vesicales, pañales, cambio de ropa y utilización de apósitos). (V)
25. In bedridden patients, do not exceed 30º in the elevation of the head. (T)	25. No sobrepasar los 30º en la elevación del cabecero de la cama en personas encamadas. (V)
26. Perform a comprehensive assessment in every patient to identify risk factors for pressure injuries. (T)	26. Realizar una evaluación completa de todos los pacientes para identificar los factores de riesgo de lesiones por presión. (V)
27. Examine the skin for signs of redness, areas of non-blanchable erythema, localized heat, induration, or skin breakdown in individuals at risk for pressure injuries. (T)	27. Inspeccionar la piel buscando signos de enrojecimiento, blanqueamiento de zonas enrojecidas, calor localizado, induración y ruptura de la piel en individuos en riesgo de lesiones por presión. (V)
28. The amount of time an individual spends sitting still does not influence the development of pressure injuries. (F)	28. El tiempo que un individuo pasa sentado sin moverse no influye en el desarrollo de lesiones por presión. (F)
29. In patients in bed in the prone position, the face, nose, chin, forehead, cheekbones, chest, knees, fingers, genitals, clavicles, iliac crest, symphysis, and back of both feet should be assessed. (T)	29. En pacientes en decúbito prono, evaluar la región de la cara, nariz, mentón, frente, pómulos, pecho, rodillas, dedos, genitales, clavículas, cresta ilíaca, sínfisis y dorso de ambos pies. (V)
30. Systematically use a validated risk assessment scale (Braden, Norton, or EMINA). (T)	30. Utilizar de forma sistemática una escala de valoración de riesgo validada (Braden, Norton o EMINA). (V)
31. In bedridden patients, monitor the skin in high-risk areas for pressure injuries (such as the heels, sacrum, occipital, nose, and hips). (T)	31. Vigilar las zonas especiales de riesgo de desarrollar lesiones por presión: talones, occipital, pabellones auditivos, nariz, pómulos y zona sacrocoxígea. (V)
